# Pitfalls of Using ANS Dye Under Molecular Crowding Conditions

**DOI:** 10.3390/ijms252413600

**Published:** 2024-12-19

**Authors:** Sergey A. Silonov, Alexander I. Kuklin, Semen V. Nesterov, Irina M. Kuznetsova, Konstantin K. Turoverov, Alexander V. Fonin

**Affiliations:** 1 Laboratory of Structural Dynamics, Stability and Folding of Proteins, Institute of Cytology, Russian Academy of Sciences, 4 Tikhoretsky Ave., 194064 St. Petersburg, Russia; silonovsa@incras.ru (S.A.S.); semen.v.nesterov@phystech.edu (S.V.N.); imk@incras.ru (I.M.K.); kkt@incras.ru (K.K.T.); 2 Frank Laboratory of Neutron Physics (FLNP), Joint Institute for Nuclear Research, 141980 Dubna, Russia; alexander.iv.kuklin@gmail.com

**Keywords:** macromolecular crowding, crowding agents, fluorescent hydrophobic dyes, ANS, protein conformational transitions, GdnHCl

## Abstract

The 1-anilino-8-naphthalenesulfonate (ANS) fluorescent dye is widely used in protein folding studies due to the significant increase in its fluorescence quantum yield upon binding to protein hydrophobic regions that become accessible during protein unfolding. However, when modeling cellular macromolecular crowding conditions in protein folding experiments in vitro using crowding agents with guanidine hydrochloride (GdnHCl) as the denaturant, the observed changes in ANS spectral characteristics require careful consideration. This study demonstrates that crowding agents can form clusters that interact differently with ANS. Furthermore, GdnHCl can disrupt these clusters and directly affect the ANS spectral characteristics. A model for the interaction between GdnHCl, crowders, and ANS is proposed. Using bovine serum albumin (BSA) as a model protein, the limitations of using ANS for studying conformational transitions induced by GdnHCl in the presence of crowding agents are demonstrated.

## 1. Introduction

Fluorescent dyes have long been widely used in biological research, particularly in studying protein structure, dynamics, conformational changes, and aggregate formation. Many dyes have specific applications. Fluorescent dyes allow marking some cellular structures and obtaining information on the structure dynamics of the stained proteins in the vicinity of the fluorescent label [1,2,3,4,5]. Typically, such dyes are solvatochromic [6,7]. Specific fluorescent probes are also used to study hydrophobic regions naturally present in certain proteins and protein-folding intermediates [8,9,10,11,12,13]. ANS is one of the most frequently used dyes for this purpose [10,14,15,16].

Free ANS has a low fluorescence quantum yield and short fluorescence excited state lifetime in aqueous solution [9]. The free ANS fluorescence decay is nearly monoexponential [17,18]. ANS can bind to both hydrophobic and charged protein regions, which results in a substantial increase in its quantum yield, whereas its fluorescence decay becomes non-monoexponential and depends on the stoichiometry of dye–protein binding [9,19,20]. The ANS transition from an almost non-fluorescent state to a state with high fluorescence quantum yield upon binding to partially folded protein intermediates has made this dye a widespread and powerful tool for studying protein folding [9,11,14,15,21,22,23].

Protein conformational studies are primarily conducted in dilute aqueous solutions. However, it is well established that the intracellular environment is characterized by limited free water, constant steric contacts between macromolecules, and minimal available volume [24,25,26,27,28]. These conditions, termed ‘macromolecular crowding’, are known to affect various biochemical processes, including protein folding, protein–protein interactions (PPI), protein complexation with various ligands and cofactors, binding with small molecules, enzymatic activity, protein aggregation, and amyloid fibril formation [24,25,26,27,28,29]. In vitro, such conditions are simulated using highly concentrated solutions of ‘inert’ polymers (‘crowding agents’) [24]. When using crowding agents, it is assumed that the polymer molecules exclusively participate in steric interactions with the studied object and do not form supramolecular structures. However, there is evidence demonstrating the ability of certain crowding agents to bind biopolymers and form cluster structures [30,31,32,33,34]. This calls into question the ‘inertness’ of crowding agents in relation to the studied objects.

Crowding agents may influence the spectral properties of fluorescent dyes [35]. While ANS has been widely used to investigate protein conformational transitions under macromolecular crowding conditions [13,29,36,37,38,39,40,41,42], the photophysical properties of ANS itself under these conditions remain understudied. Understanding these properties is crucial for accurate interpretation of protein–ANS interaction data [43]. Therefore, this work aimed to investigate the effects of crowding agents and guanidine hydrochloride (commonly used for modeling protein conformational transitions in vitro) on the ANS spectral characteristics.

## 2. Results and Discussion

### 2.1. Fluorescent Characteristics of ANS in Aqueous GdnHCl Solutions

**Figure 1:**
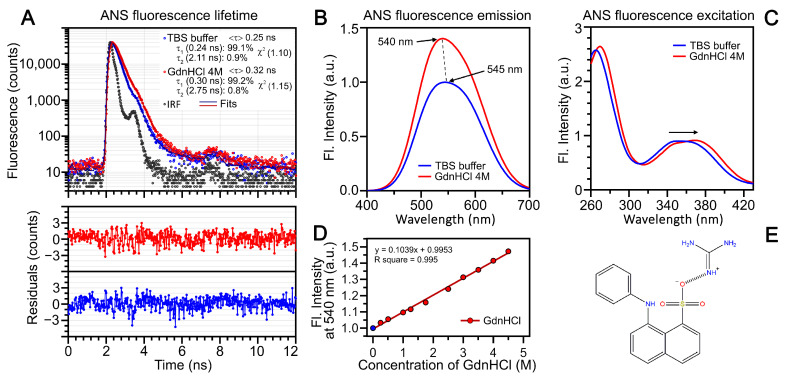
Spectral characteristics of ANS in the presence of GdnHCl. (**A**) Fluorescence lifetime measurements: ANS in Tris-HCl buffer (TBS, blue), ANS in 4 M GdnHCl solution (red), and instrument response function (IRF, black). Residuals are shown in the panel below. λex = 375 nm. (**B**) ANS fluorescence emission spectra at λex = 375 nm. Arrows show spectral maxima, with a dashed line highlighting the shift from 545 nm (TBS buffer) to 540 nm (4 M GdnHCl). (**C**) ANS fluorescence excitation spectra (λem = 540 nm), showing the shift in 4 M GdnHCl compared to the buffer solution. (**D**) Dependence of ANS fluorescence intensity at emission wavelength 540 nm on GdnHCl concentration (λex = 375 nm). (**E**) Schematic representation of proposed interaction between guanidinium ion and ANS sulfo-group.

To investigate the effect of GdnHCl on ANS fluorescent characteristics, we measured absorption spectra, fluorescence excitation spectra, fluorescence emission spectra, and fluorescence decay curves of ANS in aqueous solutions with varying GdnHCl concentrations (Figure 1).

In the presence of GdnHCl, ANS fluorescence excitation spectra exhibit a red shift (Figure 1C). At 4 M denaturant concentration, the shift is approximately 5 nm. With increasing GdnHCl concentration in the studied solutions, we observed a blue shift in the ANS fluorescence spectrum (from 545 nm to 540 nm), a 1.5-fold increase in the dye’s fluorescence intensity (Figure 1B,D), and a slight increase in the ANS excited state lifetime (Figure 1A). The ANS fluorescence decay curves in GdnHCl solutions are nearly monoexponential. The blue shift of the spectrum and increases in both fluorescence intensity and excited state lifetime of ANS may be attributed to the salt bridge formation [22] between the negatively charged ANS sulfonate group and the positively charged guanidinium ion (Figure 1E).

### 2.2. Fluorescent Characteristics of ANS in Crowding Agent Solutions

**Figure 2:**
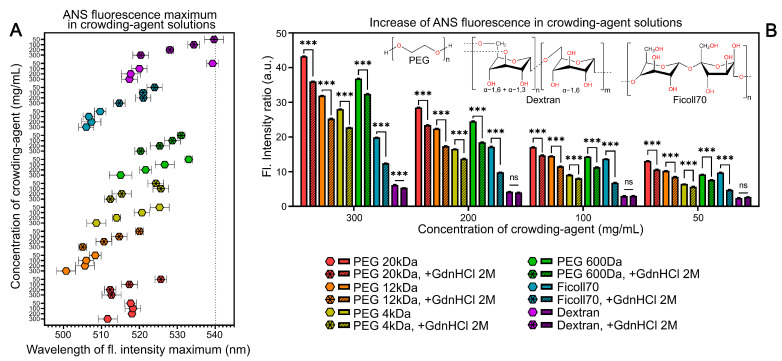
Changes in ANS fluorescence maximum and intensity in crowding agent solutions. (**A**) ANS fluorescence emission maxima at different crowding agent concentrations. The dashed line indicates the emission maximum of ANS in 4 M GdnHCl aqueous solution. (**B**) ANS fluorescence intensity in solutions with varying crowding agent concentrations. Values were normalized to ANS fluorescence intensity in TBS buffer. λex = 375 nm. Data represent mean ± SD from ≥4 independent experiments. *** *p* < 0.001, ns—not significant.

In crowding agent solutions, a significant increase in intensity and a blue shift of the ANS fluorescence spectrum are observed compared to the corresponding characteristics of the dye in aqueous solutions (Figure 2). Both the increase in ANS fluorescence intensity and the blue shift of the dye’s fluorescence spectrum persist with increasing crowding agent concentrations in the studied solutions. The effects on ANS fluorescent characteristics significantly depend on the crowding agents’ structure. Dextran, a ribbon-like glucose polymer carrying numerous hydroxyl groups [44], exhibits the least influence on the steady-state fluorescence characteristics of the studied dye. In contrast, in Ficoll 70 solutions, which is also a sugar-containing polymer, the observed spectral changes are considerably more pronounced. Ficoll’s structure (a sucrose–epichlorohydrin copolymer) represents a compact, slightly deformed sphere formed by highly branched polymer chains [44]. This might suggest that Ficoll’s more complex supramolecular structure compared to dextran accounts for the substantial difference in ANS spectral characteristics in solutions of these crowding agents, likely due to enhanced dye–polymer interactions.

The ANS fluorescent characteristics undergo significant changes in highly concentrated PEG solutions. However, no linear correlation is observed between PEG’s molecular weight and its effect on ANS (Figure 2B). For instance, the fluorescence intensity of ANS in PEG 600 solutions (polymerization degree n = 14) is higher than in PEG 4000 (n = 90) but lower than in PEG 20,000 (n = 454). It should be noted that ethylene glycol is a relatively nonpolar solvent, and PEG forms a network structure [44]. The observed differences in ANS characteristics across various PEG solutions may be attributed to both the supramolecular structures of this polymer and the proportion of free water and overall solvent polarity.

The analysis of ANS fluorescence decay curves provided essential information about its characteristics in crowding agent solutions. The decay of ANS fluorescence in PEG solutions is well approximated by a three-exponential dependence. The lifetime of the shortest-lived ANS fluorescence component (τ = 0.3–0.7 ns) is close to the excited state lifetime of free ANS in aqueous solutions (Table S1). While in PEG solutions at 100 mg/mL (~10% aqueous solution), regardless of the polymer molecular weight, this component contributes 30–40% to the total fluorescence, in PEG solutions at 300 mg/mL (~30% aqueous solution) its contribution to the total fluorescence does not exceed 2–4%. This decrease in the short-lived component indicates a reduction in the fraction of free water in PEG solutions as the concentration of crowding agents increases. The lifetime of the long-lived ANS fluorescence component (τ = 4.0–5.5 ns) matches the excited state lifetime of this dye in ethylene glycol solutions (Figure S1, Table S1), which serves as a reference for nonpolar environments. This may indicate ANS localization in hydrophobic PEG clusters. The contribution of this component is insignificant and does not exceed 7% in all studied PEG solutions.

The major contribution to the total ANS fluorescence comes from the component with a characteristic lifetime of 0.9–1.5 ns (Table S1). The contribution of this component changes inversely with the contribution of the short-lived ANS component (τ = 0.3–0.7 ns) as the PEG concentration increases in the studied solutions. We suggest that ANS molecules with such excited state lifetimes interact with PEG, specifically with its ether oxygen atoms [45].

The ANS decay curves in Ficoll and dextran solutions require approximation by four -exponential dependencies. The shortest-lived ANS fluorescence components (τ = 0.3–0.8 ns) in Ficoll and dextran solutions apparently correspond to free ANS, while components with longer lifetimes are likely due to the dye’s interaction with these polymer molecules. The time-resolved characteristics of ANS in Ficoll solutions remain practically independent of this crowding agent’s concentration, whereas, in dextran solutions, the contribution of short-lived components to total ANS fluorescence decreases from 82% to 58% as dextran concentration increases from 50 to 300 mg/mL. Collectively, these data indicate that dextran is the most ‘inert’ crowding agent. The steady-state fluorescence characteristics of ANS in dextran solutions show minimal variation with different concentrations, and according to time-resolved spectroscopy data, these solutions exhibit the highest relative content of ‘free’ ANS molecules among all studied polymers. These properties likely arise from dextran’s linear structure, though the specific mechanism requires further investigation. For time-resolved measurements, however, Ficoll proves more suitable as an experimental system since the time-resolved characteristics of ANS in Ficoll solutions remain stable across different concentrations.

The addition of GdnHCl to the studied solutions causes a decrease in ANS fluorescence intensity (except for dextran solutions in the 50–200 mg/mL concentration range) and, in some cases, a red shift in the ANS fluorescence spectrum (Figure 2). However, the time-resolved fluorescence characteristics of ANS in crowding agent solutions show different dependence on GdnHCl (Table S1). The GdnHCl has the greatest effect on Ficoll. These observed effects are opposite to the influence of GdnHCl on the steady-state fluorescence characteristics of ANS in aqueous solutions, where GdnHCl typically causes an increase in fluorescence intensity. Given that GdnHCl is a chaotropic agent known to disrupt molecular ordering in solution, we hypothesize that the decrease in ANS fluorescence intensity in crowding agent solutions upon GdnHCl addition is due to the disruption of supramolecular structures of the studied polymers.

### 2.3. SANS Analysis of GdnHCl-Induced Structural Changes in PEG Solution

**Figure 3:**
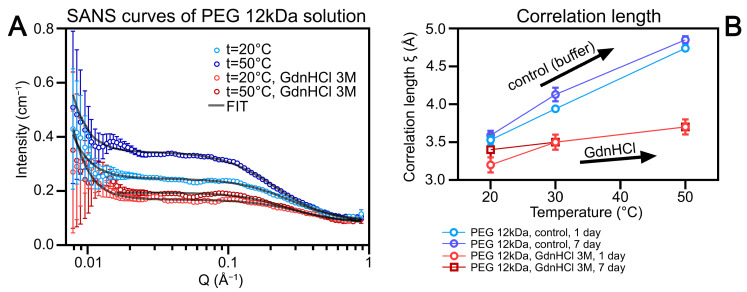
Small-angle neutron scattering (SANS) analysis of PEG 12,000. (**A**) SANS curves of PEG 12,000 in D_2_O solutions. Measurements were performed at different temperatures (20 °C and 50 °C; dark and light blue) and with or without 3 M GdnHCl (dark and light red). The fitted curves are shown in gray. (**B**) Correlation length of PEG 12,000 solutions measured after different incubation times (1 and 7 days), with or without 3 M GdnHCl.

To elucidate the mechanism of GdnHCl’s influence on crowding agent solution structure, we investigated the structure of PEG 12,000 in the presence of GdnHCl using small-angle neutron scattering (SANS). The obtained SANS curves for PEG 12,000 are shown in Figure 3. Under all experimental conditions, we could not reliably characterize PEG cluster scattering due to high uncertainty in the low Q-value region. However, the obtained data allowed reliable determination of the polymer chain correlation length, enabling characterization of the polymer gel mesh size and polymer chain packing density.

The data indicate that sample aging (7-day incubation) within the studied time range has virtually no effect on PEG structure under all experimental conditions. An increase in temperature of PEG samples without GdnHCl causes enhanced scattering in the low Q region and increased polymer chain correlation length, indicating gel swelling. The addition of GdnHCl to the PEG solution induces polymer chain collapse, confirmed by decreased scattering in the low Q region and decreased correlation length. Moreover, temperature increase in PEG samples containing GdnHCl has substantially less effect on the polymer structure compared to GdnHCl-free PEG samples. The data demonstrate that the structure and properties of 300 mg/mL PEG 12,000 solutions differ significantly in the absence and presence of 3 M GdnHCl. Thus, GdnHCl disrupts the mesh structure of PEG.

### 2.4. Fluorescent Characteristics of ANS in BSA Solutions with Crowding Agents

**Figure 4:**
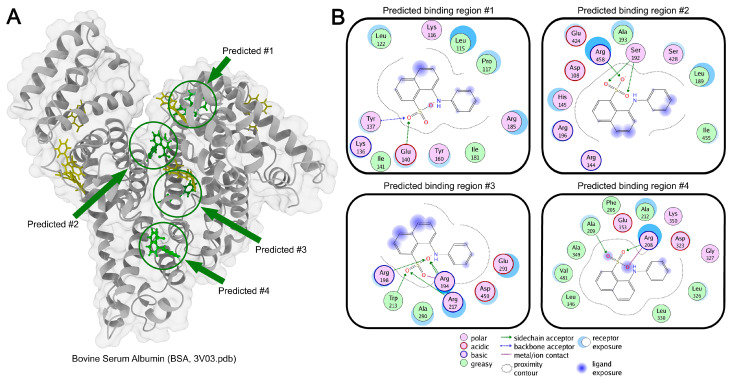
Molecular docking analysis of ANS binding to bovine serum albumin (BSA). (**A**) Three-dimensional structure of BSA highlighting predicted ANS binding sites. Four high-probability binding sites are indicated in green and highlighted by circles and arrows, while ANS molecules in six low-probability sites are shown in yellow (probability scores are provided in Supplementary Table S2). (**B**) Two-dimensional interaction map showing key amino acid residues and molecular interactions within the highest-probability ANS binding pockets.

Based on the obtained results, it can be hypothesized that when studying protein conformational transitions in crowding agent solutions using fluorescence, the solvent’s effect on the ANS signal may be comparable to the useful protein-induced signal changes. Therefore, we investigated the influence of crowding agent solutions at various concentrations on the fluorescent characteristics of ANS in the presence of 0.3 mg/mL of the model bovine serum albumin (BSA) protein. In BSA solutions at this concentration, the ANS fluorescence intensity is approximately 80 times higher than that of free ANS in aqueous solution. While ANS fluorescence in aqueous solutions exhibits monoexponential decay characteristics, BSA-bound ANS exhibits multiexponential decay. It is known that BSA has multiple binding sites for various small molecules [46]. Previous studies have also shown that ANS can bind to BSA [12,23]. It can be hypothesized that there are several ANS binding sites that differ in their microenvironment and solvent accessibility, resulting in different time-resolved characteristics for these ANS molecules. To investigate this further, a molecular modeling experiment was performed to simulate the binding of ANS molecules with BSA. According to the obtained intermolecular energy values, ANS can potentially interact with BSA through at least four sites (Figure 4). Sites #1, 2, and 3 exhibit relatively polar characteristics, and ANS molecules interacting with these BSA regions likely correspond to fluorescence components with characteristic times of 3–10 ns. In contrast, the microenvironment of site #4 is sufficiently nonpolar, and ANS molecules at this location likely correspond to fluorescence components with characteristic times exceeding 10 ns.

**Figure 5:**
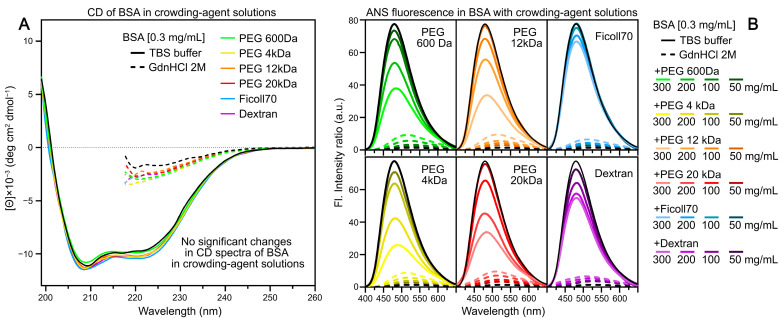
Comparison of BSA circular dichroism spectra and ANS fluorescence intensity in solutions containing crowding agents. (**A**) CD spectra of BSA (0.3 mg/mL) in buffer solutions (with and without GdnHCl) and in the presence of crowding agents (300 mg/mL). (**B**) ANS fluorescence spectra in BSA solutions with various crowding agents. Fluorescence intensity is expressed relative to ANS fluorescence in buffer solution without BSA. Dashed lines represent solutions containing 2 M GdnHCl.

The addition of crowding agents to the BSA solution results in a decrease in ANS fluorescence intensity, regardless of the crowder structure (Figure 5, Table S3). Increasing the crowding agent concentration leads to a further reduction in ANS fluorescence intensity in BSA solutions. According to far UV circular dichroism data, the BSA secondary structure remained practically unchanged in crowding agent solutions. As it is known, crowders can affect protein structure and stability differently [26,28,37,47,48,49,50,51]. However, crowding agents have a slight impact on the structure of globular proteins such as BSA [47,50,52,53,54]. The obtained data suggest that crowding agents compete with BSA for ANS binding. This hypothesis is supported by the decreased contribution of long-lived fluorescence components characteristic of ANS in aqueous BSA solutions to the total dye fluorescence in crowding agent solutions. It is known that crowding agent effectiveness depends on the ratio between the hydrodynamic radii of the polymer and target molecules. The ratio between the hydrodynamic radii of Ficoll-70 (40 Å) and BSA (34 Å) is close to 1, suggesting that this crowding agent is the most ‘inert’ in BSA solutions. Previous studies have shown that repulsion is the predominant interaction between BSA and Ficoll molecules [55]. These data explain the minimal changes in ANS steady-state fluorescence characteristics observed in BSA solutions containing Ficoll-70 compared to solutions with other crowding agents.

To further investigate the role of crowding agents, we compared their effects alone versus in combination with a chemical denaturant. The ANS fluorescence intensity in aqueous BSA solutions with 2 M GdnHCl was significantly lower than in corresponding crowding agent solutions. While BSA maintained greater secondary structure integrity in crowding agent solutions compared to aqueous solutions with 2 M GdnHCl, time-resolved fluorescence spectroscopy revealed that the contribution of long-lived components (corresponding to BSA-bound ANS) to total ANS fluorescence was substantially lower in solutions containing both polymers and GdnHCl compared to aqueous GdnHCl solution. These results indicate that the elevated ANS fluorescence intensity observed in crowding agent and GdnHCl solutions (relative to aqueous GdnHCl solutions) stems from direct interactions between ANS molecules and the polymer molecules rather than from changes in BSA structure.

## 3. Material and Methods

### 3.1. Chemical Compounds and Solution Preparation

The following crowding agents were used: polyethylene glycol (PEG) with molecular weights of 600 Da (polymerization degree n = 14), 4000 Da (n = 90), 12,000 Da (n = 270), and 20,000 Da (n = 454); Ficoll-70 with a molecular weight of 70,000 Da; and Dextran-70 with a molecular weight of 70,000 Da. Crowding agents (all from Sigma, St. Louis, MO, USA) were used without additional purification. The hydrophobic dye 1-anilino-8-naphthalenesulfonate in powder form (Sigma, St. Louis, MO, USA) and ultra-pure guanidine hydrochloride (Nacalai Tesque, Kyoto, Japan) were used without additional purification. Deionized water “Simplicity UV” was from MilliQ (Merck Millipore, Burlington, MA, USA). Powdered Tris and 1 M hydrochloric acid solution (Sigma, St. Louis, MO, USA) were used to prepare buffer solutions. TrisHCl buffer with pH 7.4 was used as the base solution. ANS concentration was determined spectrophotometrically using a Hitachi U-3900 spectrophotometer (Hitachi, Chiyoda, Japan) and molar extinction coefficient ε_372_ = 7800 M^−1^ cm^−1^. Solution mixing was performed using a “VortexV1 plus” shaker (BioSan, Riga, Latvia) and “S-4” (Elmi, Riga, Latvia). Viscous liquids were dispensed using positive displacement pipettes “MR-100” and “MR-1000” (Mettler Toledo, Greifensee, Switzerland).

### 3.2. Circular Dichroism

Circular dichroism (CD) spectra measurements were conducted using a J-810 spectropolarimeter (Jasco, Hachioji, Japan) in the far UV region (260–190 nm), performed with 0.1 nm steps using quartz cuvettes with an optical path length of 1 mm. The measurements were made at 25 °C. Background spectra containing buffer at the same composition were subtracted from all sample spectra to obtain corrected spectra.

### 3.3. Fluorescence Steady-State Experiments

The fluorescence experiments were carried out using a Cary Eclipse fluorescence spectrometer (Agilent, Mulgrave, Australia). The measurements were made at 25 °C with cells 10 × 10 mm (Starna, Atascadero, CA, USA). The fluorescence intensity of ANS was corrected to the primary inner filter effect according to [56]:

(1) $$F_{0}\left(\lambda_{ex}\right)=\frac{F\left(\lambda_{ex}\right)}{W},$$

where *W* is the factor that corrects the measured total fluorescence intensity for the so-called primary inner filter effect. Because the fluorescence measurements were performed using the Cary Eclipse spectrometer with horizontal slits, the value of correction factor *W* was calculated based on the ratio:

(2) $$W=\frac{\left(1-10^{-A_{\Sigma }}\right)}{A_{\Sigma }},$$

where *A_Σ_* is the total absorbance of exciting light in the solution.

The excitation wavelength for the ANS fluorescence was 350 nm. The emission wavelength for the dye fluorescence ranged from 400 to 700 nm. The excitation fluorescence spectra of ANS were recorded at an emission wavelength of 540 nm.

### 3.4. Time-Resolved Fluorescence Measurements

Time-resolved fluorescence measurements were carried out by a time-correlated single-photon counting approach using spectrometer Fluotime 300 (PicoQuant, Berlin, Germany) with Laser Diode Head LDH-375 (*λ_ex_* = 375 nm). Measured fluorescence intensity decays were fit to a multi-exponential model

(3) $$F\left(t\right)=\int_{-\infty }^{t}IRF\left(t\prime \right)\sum_{i=1}^{n}\alpha_{i}\cdot \exp\left(-\frac{t-t\prime }{\tau_{i}}\right)dt\prime ,$$

where *α_i_* is the amplitude and *τ_i_* is the lifetime of *i*th decay component, IRF is the instrument response function. The convolution of the model exponential function with the IRF was compared to the experimental data until a satisfactory fit was obtained. The IRF was measured using a cross-correlation of the excitation and fundamental gate pulse. The FluoFit version 4.5 software (Pico Quant, Germany) was used for the analysis of decay curves.

### 3.5. Molecular Docking

Molecular modeling calculations and docking studies were performed using AutoDock Vina (v. 1.1.2) [57] and Molecular Operating Environment (MOE, v.2014.0901) [58] software (Chemical Computing Group Inc., Montreal, QC, Canada). The X-ray crystallographic structure of bovine serum albumin (BSA) was obtained from the RCSB Protein Data Bank (PDB ID: 3V03).

### 3.6. Small-Angle Neutron Scattering (SANS)

PEG 12000 solutions with a concentration of 300 mg/mL were additionally characterized using small-angle neutron scattering. Measurements were performed using the YuMO facility at the IBR-2 reactor (JINR, Dubna, Russia) at two detector systems [59,60]. To increase contrast in the analyzed samples, PEG 12,000 was dissolved in D_2_O (Sigma, USA). Considering that in 300 mg/mL PEG 12,000 solutions, hydrogen atoms in PEG constitute 24.6% of the total hydrogen atoms in the solution, solutions with corresponding H_2_O and D_2_O ratios were prepared for correct background scattering accounting. To determine GdnHCl’s effect on PEG structure in the studied solutions, D_2_O-based solutions containing 300 mg/mL PEG 12,000 and 3M GdnHCl were prepared. For background scattering accounting in such systems, 3 M GdnHCl solutions based on D_2_O and H_2_O were prepared. Measurements were performed in “Hellma” cuvettes [61] with optical path lengths of 1 and 2 mm at temperatures of 20, 30, and 50 °C. Primary SANS data processing included corrections for sample transmission and thickness, as well as electronic noise using the SAS program [62]. Wavelength calibration using silver behenate was performed according to [63]. PEG small-angle scattering curves were processed using the SasView version 5.0 software package and characterized by the following functional [64]:

(4) $$I\left(Q\right)=\frac{A}{Q^{n}}+\frac{C}{1+{\left(Q\xi \right)}^{m}}+Bkg,$$

where *I(Q)* is scattering intensity, *Q* is the scattering vector modulus, *Bkg* is background scattering, *A*, *C*, *n*, and *m* are approximation parameters, and *ξ* is the correlation length of polymer chains. The first term of this functional determines polymer cluster scattering, while the second term is a Lorentzian and characterizes the interaction between polymer chains and solvent.

## 4. Conclusions

Investigation of spectral, fluorescent, and small-angle neutron scattering characteristics of ANS in crowder–GdnHCl systems revealed significant limitations in using the ANS fluorescent dye for studying protein conformational transitions under macromolecular crowding conditions. Multiexponential ANS fluorescence decay demonstrates heterogeneous clustering of crowding agents in solution. Significant differences in ANS fluorescent characteristics in bovine serum albumin (BSA) solutions (0.3 mg/mL) with different crowding agents indicate possible interactions between crowding agent molecules and BSA. Among all studied crowding agents, Ficoll-70 demonstrated the least interaction with BSA, making it the most suitable crowding agent for such studies. Computer modeling revealed four possible ANS binding sites on BSA, of which three sites showed partial solvent accessibility for the ANS molecule.

Thus, studying protein folding–unfolding processes in the presence of crowding agents using ANS fluorescent dye is complicated by two factors: the dye’s interaction with crowding agents and its interaction with the chemical denaturant GdnHCl, both of which significantly affect its spectral characteristics. Moreover, the GdnHCl–crowder solution represents a complex system whose structure and interaction with proteins depend on the concentrations of both denaturant and crowder. These factors significantly complicate the planning and execution of control experiments. Consequently, interpretations of in vitro protein folding–unfolding studies under simulated cellular crowding conditions should be approached with great caution.

## Data Availability

The original contributions presented in this study are included in the article/Supplementary Material. Further inquiries can be directed to the corresponding author.

## Supplementary Materials

The following supporting information can be downloaded at https://www.mdpi.com/article/10.3390/ijms252413600/s1.
