# Supplementary material for: Pitfalls of Using ANS Dye Under Molecular Crowding Conditions
Source: Int J Mol Sci. 2024 Dec 19;25(24):13600. doi: 10.3390/ijms252413600 (PMC11676346; doi:10.3390/ijms252413600)
Supplement: Supplementary file 1 [file ijms-25-13600-s001.zip › Figure S1. Fluorescence characteristics of ANS in different solvents.pdf]

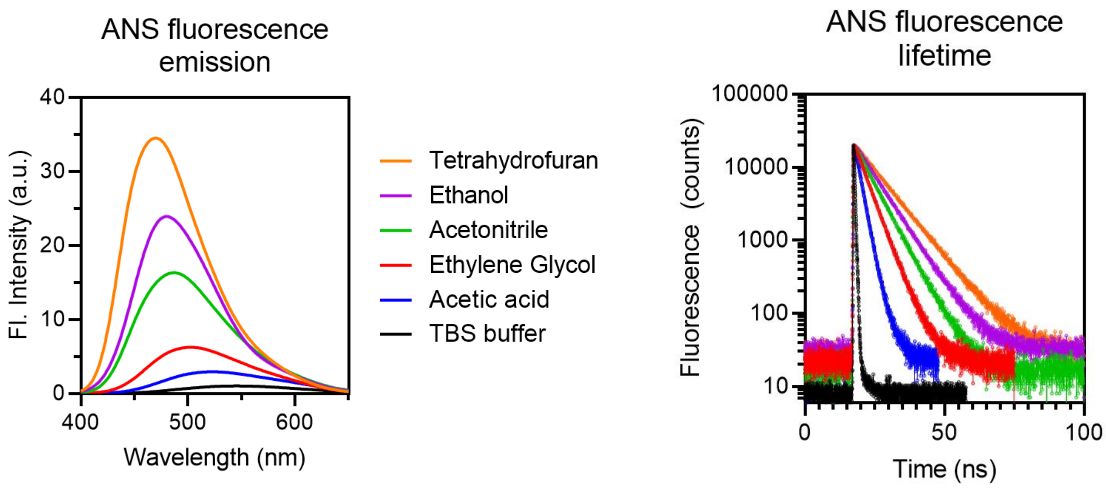

Solvent	Imax, nm	<τ>, ns	χ2
Tris-HCl (TBS, pH 7.4)	545	0.248	1.103
Acetic acid (17.5M)	525	1.715	1,160
Ethylene Glycol	504	4.231	1,085
Acetonitrile	487	6.008	1,119
Ethanol (95%)	480	7.298	1,094
Tetrahydrofuran	470	9.101	1,045

Figure S1. Fluorescence characteristics of ANS in different solvents. (**Top left panel**) ANS fluorescence emission spectra normalized to the maximum intensity in TBS (Tris-HCl) buffer. (**Top right panel**) Fluorescence decay curves. (**Bottom panel)** Summary of maximum fluorescence intensities and calculated fluorescence lifetimes.
